# The seismic waveform dataset of the Sardinia Passive Array Experiment (SPAE)

**DOI:** 10.1016/j.dib.2019.103927

**Published:** 2019-04-17

**Authors:** Giovanni Battista Cimini

**Affiliations:** Istituto Nazionale di Geofisica e Vulcanologia, Italy

**Keywords:** Sardinia, Temporary seismic network, Earthquake monitoring, Waveform dataset, EIDA

## Abstract

The Sardinia Passive Array Experiment (SPAE) was carried out in central-northern Sardinia (Italy), between July 2014 and September 2016, with the main goal of acquiring new data for studies on the lithospheric structure of Mediterranean region and seismicity of the Sardinia-Corsica block. The array consisted of 8 three-component portable seismographs, deployed progressively through the observing period. The stations operated in continuous mode recording at 100 sps, providing more than 50 GB of digital waveforms for the analysis. This data article describes the main characteristics of the temporary network and the valuable content of the seismic dataset assembled. The SPAE waveform dataset and the station metadata are accessible at the European Integrated Data Archive (EIDA) repository.

**Table:** Specifications Table

Subject area	*Geophysics*
More specific subject area	*Seismology*
Type of data	*Tables, figures, text file, digital time-series.*
How data was acquired	*Seismic campaign using a temporary network of mobile seismographs composed by high dynamic digitizers and three-component extended band or broad band seismometers.*
Data format	*Raw, filtered, partially processed.*
Experimental factors	*The passive experiment was conducted with the seismic stations in continuous mode recording at a sampling rate of* 100 Hz.
Experimental features	*Raw data were acquired at each station for at least three months.*
Data source location	*Sardinia (Italy).*
Data accessibility	*With this article (station data, PDF plots, script) and on**https://www.orfeus-eu.org/data/eida/*(waveform dataset).
Related research article	*G.B. Cimini, A. Marchetti, M. Silvestri, L’esperimento Sardinia Passive Array (SPA): acquisizione dati sismici per lo studio della geodinamica e della sismotettonica dell’area mediterranea, INGV Technical Reports 334 (2016) 28 pp.*http://hdl.handle.net/2122/10270*(in Italian)**[1]**.*

**Table:** 

**Value of the data** • Can be integrated with the data collected by other networks for new seismological studies of the Central-Western Mediterranean. • Can be used to improve the pattern of seismicity of the Sardinia-Corsica continental block. • The teleseismic component of the dataset is pivotal for modelling the deep structure beneath the region by applying advanced techniques like Receiver function, 3-D Tomography. • The long-term ambient noise record can provide useful information to site selection process for further temporary deployments and extension of Italian permanent network in the island. • The whole data set is exported to EIDA in a standard miniSEED data format.

## 1 Data

The data described in this article consist of a collection of seismic waveforms acquired during the Sardinia Passive Array Experiment (SPAE) carried out in the period July 2014–September 2016. Eight sites in the central-northern part of Sardinia Island (Italy) were monitored through the survey using high dynamic, portable instrumentation. Table 1 summarizes the station characteristics and duration of recording at each site. Sardinia is located in the middle of the Central-Western Mediterranean (CWM) and, together with Corsica, forms a 30-35 km-thick crustal block [2] bounded by two oceanic basins (the Tyrrhenian basin to the East and the Liguro-Provençal basin to the West) affected by extensional tectonics [3]. Novel seismic data from this location are fundamental for new, high-resolution, tomography studies of the upper mantle structure beneath the CWM [4], as well as for a better characterization of the seismicity pattern of Sardinia, popularly considered to be the only aseismic region of Italy.

**Table 1:** Station data of the Sardinia Passive Array Experiment. Site geology is from Ref. [3].

Station code	Location			Site geology	Data acquisition	
	Lat. (N)	Lon. (E)	Ele. (m)		Start	End
SPX1	40.82101	9.10094	855	Leucogranites	2014-07-17	2015-05-27
SPX2	40.44266	8.53796	644	Ignimbrites	2014-07-16	2016-09-27
SPX3	39.87674	9.50299	1131	Metavolcanic rocks	2014-07-17	2015-04-28
SPX4	40.73579	8.28031	93	Conglomerates	2014-12-10	2015-03-17
SPX5	40.69413	9.57943	751	Leucogranites	2015-04-29	2016-07-26
SPX6	40.58226	8.73193	311	Marly sandstones	2015-07-15	2016-02-17
SPX7	40.02666	8.93322	451	Tonalites	2016-02-17	2016-05-25
SPX8	40.52855	9.04376	659	Metasandstones	2016-05-26	2016-09-27

The seismic campaign was performed with an average of three stations recording at the same time. This limited deployment of simultaneously active stations has provided nevertheless a substantial increase in the number of seismographs covering the area of the Sardinia-Corsica block. In fact, before the experiment, only three stations were active in Sardinia (Table 2) and four in Corsica, the latter belonging to the French seismological network (RESIF). Data from the operational sites during the SPAE acquisition period can be integrated to study not only the seismicity of the region (initial results on this topic are presented in Ref. [1]), but also the crustal structure of the block by means, for instance, receiver function techniques which use teleseismic wave records at a single station. For such investigations, the SPAE dataset can potentially provide the waveforms of over 250, Mw > 5.5, teleseisms occurred worldwide.

**Table 2:** Permanent stations of the Italian National Seismic Network in Sardinia.

Station code	Location			Sensor Type BB = Broad Band VBB = Very Broad Band	Start-date	Status
	Lat. (N)	Lon. (E)	Ele. (m)			
AGLI	41.127	9.173	180	BB – 40s	2017-03-21	Open
CGL	39.366	9.296	1050	BB – 20s	2003-03-01	Open
DGI	40.318	9.607	354	BB – 40s	2003-03-01	Open
SU26	39.123	8.490	66	BB – 120s	2017-03-06	Open
VSL	39.496	9.378	370	VBB – 360s	1989-07-19	Open

## 2 Experimental design, equipment and data assembly

### 2.1 Experimental design

**Fig. 1:**
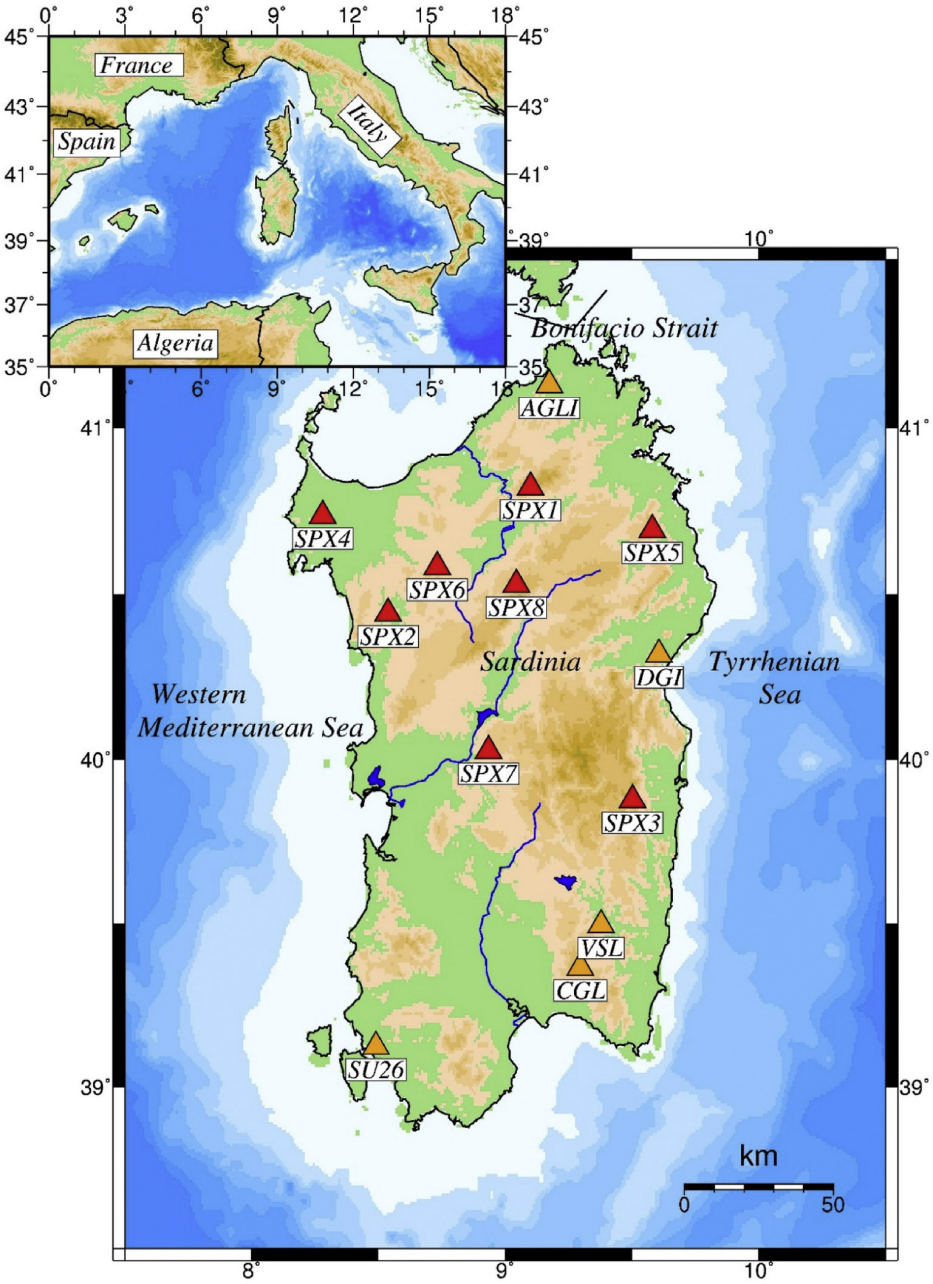
Map of Sardinia Island with the SPAE stations (red triangles). Orange triangles are the permanent stations of the Italian National Network. Insert map shows the Central-Western Mediterranean area around the Sardinia-Corsica block.

The layout of the passive experiment was primarily designed to increase, especially in the western part of Sardinia, the station coverage provided by the Italian National Seismic Network (RSN) managed by the Istituto Nazionale di Geofisica e Vulcanologia (INGV). The basic data of the permanent installations are given in Table 2. Fig. 1 displays the SPAE temporary stations and the current distribution of the RSN network. During the observing period only three permanent stations were active as stations AGLI and SU26 have been installed later. The permanent seismographs are almost all located in the eastern part of Sardinia, along the Tyrrhenian Sea side. This distribution took place over the last decades mainly to obtain more constrained hypocenter locations of the Apennines and southern Tyrrhenian seismicity. As a secondary goal, the SPAE project aimed to improve such geometry nowadays clearly inadequate. In particular, the seismic monitoring at SPX8 site, one of the quietest sites of the temporary array, provided important data for a long-term background noise investigation conducted later in the surrounding area [5], [6]. This extension of SPAE completed the site selection for a new permanent broad-band station, which is currently under construction.

### 2.2 Equipment

The mobile stations consisted of RefTek RT130, three channels, 24 bit ADC data loggers (https://www.reftek.com) and mainly Lennartz 3D/5s extended-band seismometers (www.lennartz-electronic.de). A Trillium Compact 120s broadband seismometer (https://www.nanometrics.ca) was also installed, initially at the monitoring site SPX2 then at the more suitable site SPX1 (Table 3). An external Garmin GPS Receiver provided the time for the internal clock synchronization and position reference. The power supply was made by a 12V battery connected to a 50/70W solar panel. Acquisition mode was set to continuous with a sample rate of 100 sps, preamplifier gain set to 1, and compressed data format for the storage. The digital time series were recorded on an hourly basis and downloaded on two Compact Flash (CF) cards having 2 GB capacity each. With these configuration parameters, every station collected about 30 MB of raw data *per* day, on average.

**Table 3:** Site location and instrumentation of SPAE stations.

Station	Locality and recording site	Instrumentation	
		Digitizer	Sensor
SPX1	Oschiri - *S'Ampulla* Mountain Colony	RT130	LE3D/5s from 07/14 to 03/15 Trillium 120s from 03/15 to 05/15
SPX2	Villanova - *Mt. Minerva* State Forest	RT130	Trillium 120s from 07/14 to 03/15 LE3D/5s from 03/15 to 09/16
SPX3	Lanusei - *Mt. Armidda* Astronomical Observatory	RT130	LE3D/5s
SPX4	Sassari - *La Corte* Primary School	RT130	LE3D/5s
SPX5	Torpè - *Usinavà* State Forest	RT130	LE3D/5s
SPX6	Siligo - *Siligo* Astronomical Observatory	RT130	LE3D/5s
SPX7	Ula Tirso - *St. Isidoro* Country Church	RT130	LE3D/5s
SPX8	Bultei - *Fiorentini* State Forest	RT130	LE3D/5s

**Fig. 2:**
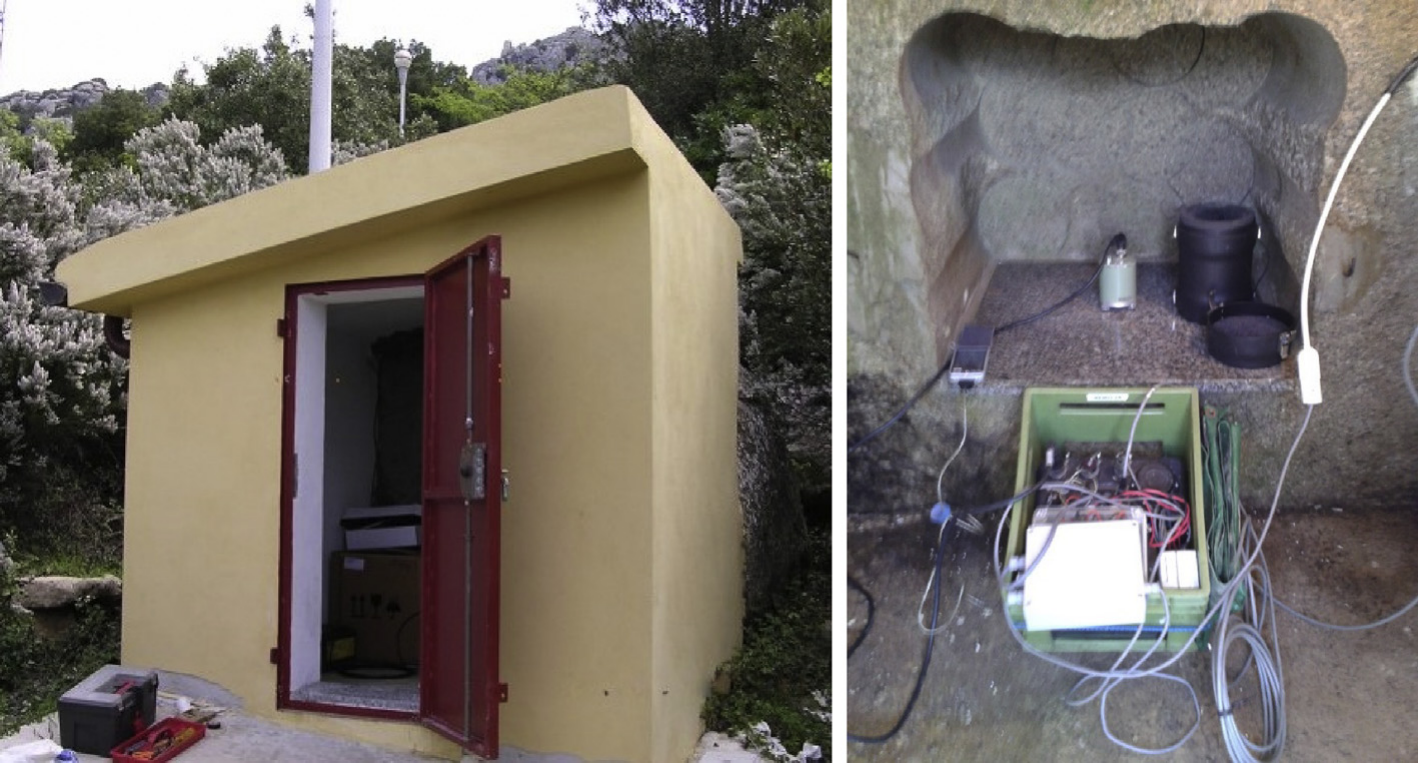
SPX1 station site (left) and instrumentation installed (right). The station was located on the western slope of Mount Limbara (Carboniferous-Permian plutonic complex). In the first part of the experiment the station operated with RefTek RT130 recorder (in the plastic green box) and Lennartz 3D/5s seismometer then, starting from March 2015, with Trillium Compact 120s seismometer (in the granite cavity).

**Fig. 3:**
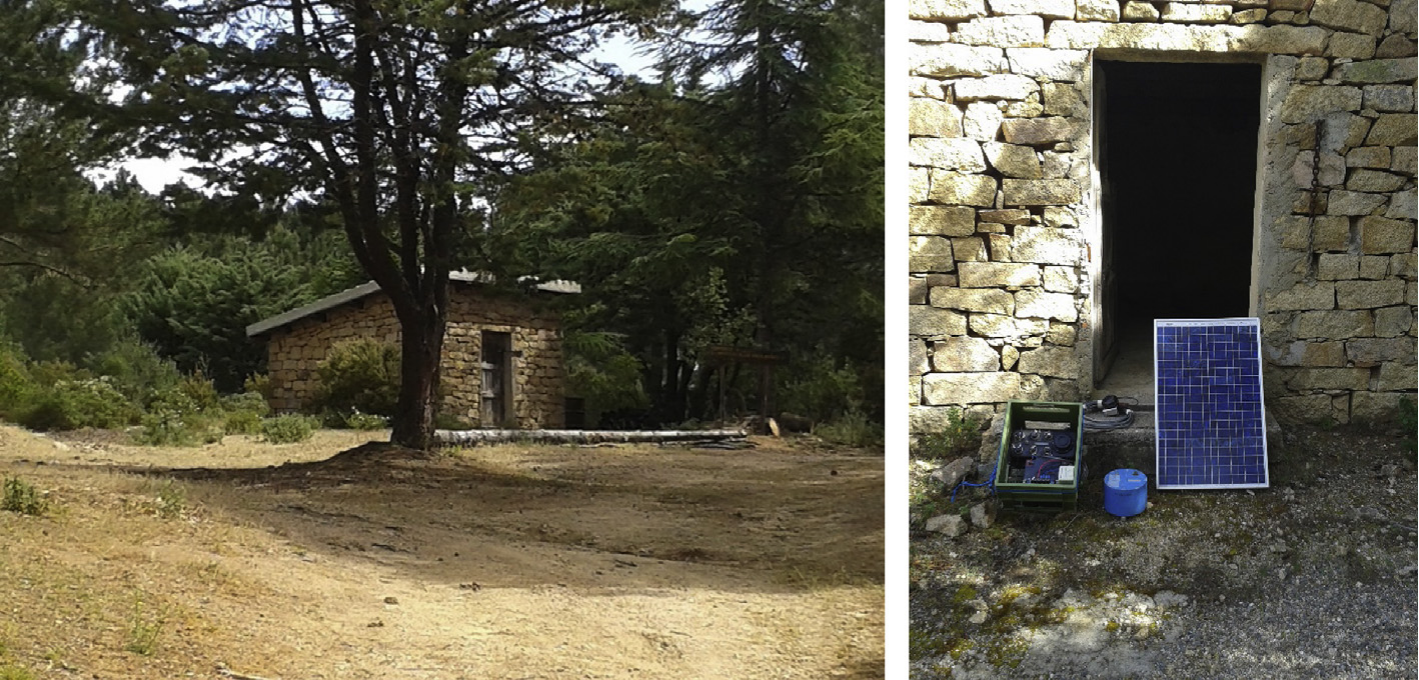
SPX5 station site (left) and instrumentation installed (right). The station was located in a remote state forest and equipped with RefTek RT130 recorder and Lennartz 3D/5s seismometer.

Fig. 2, Fig. 3 show pictures of some SPAE station sites, mostly consisting of small safe buildings located as far away as possible from the common sources of anthropic noise [7], along with the seismological equipment deployed within them. The overall quality of each station was estimated by examining the distribution of background noise power spectral density (PSD) within different time intervals. The analysis was performed by using the noise processing system (PDF Analysis) described in Ref. [5]. PDF noise plots for each channel of the SPAE seismometers are attached as supplementary material (Appendix A). The diagrams show the PSD samples computed for a week-long recording period taken mainly in winter time. The colored vertical bar on the right expresses the probability of occurrence of a given power at a particular frequency. Details on the probability density function (PDF), on the reference high (HNM) and low (LNM) noise models, and on the main sources of noise and signals characterizing the PDFs can be found in Ref. [5]. In general, the ambient noise conditions, mapped as high probability of occurrence, are good at all the SPAE temporary stations. Particularly for SPX2, SPX5, SPX6, and SPX8, the noise levels exhibit limited variations moreover across a wide range of frequencies (from about 0.1 to at least 10–20 Hz).

### 2.3 Data assembly

The RefTek 130 Data Acquisition Systems (DAS units) store data in packets of 1020-bytes each. Each packet contains a 16-byte header followed by a 1008 bytes section allocated differently according to the packet type. Examples of packet type codes are DT (Data Packet), SH (State-Of-Health Packet), SC (Station/Channel Parameter Packet). Files are written in FAT32 format and usually compressed to allow long acquisition periods to be stored on CF cards. For instance, the two 2-GB memory cards installed in the SPAE data loggers provided enough storage to hold more than 120 days of three channel 100 sps data recorded with Steim-2 compression.

The above RefTek proprietary raw format can be converted in seismic data formats as SEG-Y, SAC, mini-SEED, and others, using the pre-built PASSOFT software package available at the PASSCAL Web-site http://www.passcal.nmt.edu/content/software-resources. PASSCAL is the Portable Array Seismic Studies of the Continental Lithosphere program of the Incorporated Research Institutions for Seismology (IRIS) consortium (https://www.iris.edu/hq/programs/passcal).The SEG-Y file format is one of the several standards developed by the Society of Exploration Geophysicists (SEG) for storing geophysical data [8]. SAC data files are the standard input of the Seismic Analysis Code (https://ds.iris.edu/files/sac-manual/sac_manual.pdf), which is a general-purpose interactive program widely used for the study of sequential signals, especially time-series data. The mini-SEED format is a stripped down version of the SEED (Standard for the Exchange of Earthquake Data) format which is the international standard format for the exchange of digital seismological data. In current usage, SEED formatted files contain the digital time series data and meta information such as station and channel ID, sample rate and instrument response [9]. Mini-SEED files mainly contain waveform data, dataless SEED files only metadata. Extensive information on the format can be found in SEED Reference Manual [10] available at the Federation of Digital Seismographic Networks (FDSN) Web-site (https://www.fdsn.org/seed_manual/).

Both mini-SEED files and station metadata have been created for the SPAE seismic dataset, according to the requirements for uploading data into the EIDA archive (https://www.orfeus-eu.org/data/eida/). The mini-SEED files were generated with a Bash shell script running the PASSOFT program *rt2ms*, which converts RT130 raw data to mini-SEED. As an example, the command line

rt2ms –f home/SPAE/SPX1/2014/2014365 –p SPX1.par —R

takes the RT130 files (-f) collected by the station SPX1 in the last day of 2014 (2014365) and write them in mini-SEED format into a subdirectory (-R) named R365.01. The –p option allows to specify a parameter file containing the basic information about the station, channels, and acquisition mode. The program uses these data to modify the headers, and to specify the byte order, block size and encoding. The parameter file for SPX1 matches the scheme below.

#das; station; refchan; refstrm; netcode; channel; encoding; samplerate; gain

932F; SPX1; 1; 1; 5J; EHZ; STEIM2; 100; 1

932F; SPX1; 2; 1; 5J; EHN; STEIM2; 100; 1

932F; SPX1; 3; 1; 5J; EHE; STEIM2; 100; 1

Fields *refstrm* and *netcode* are respectively the data stream used (velocity) and the code assigned by the FDSN to the temporary network. The channels name are according to the Seismic Channel Naming convention (SEED Reference Manual - Appendix A; [10]). The first letter specify the response band of the instrument and the sampling rate (letter E stand for an Extremely Short Period with corner period <10s and sample rate in the range ≥80 to <250 Hz), the second specify the family to which the sensor belongs (H, High Gain Seismometer), and the third letter is the orientation code (Z, vertical; N, North-South; E, East-West). In the last part of the experiment station SPX1 operated with a Trillium Compact 120s seismometer, hence the channels name was set to HH*, indicating a High Broad Band sensor with a corner period ≥10s.

The Unix script that performs the data conversion for a single station is given as supplementary material (Appendix B). In the code, variables MSDIR# and MSF# define the data directory structure and the name of the mini-SEED files according to the SeisComP Data Structure (SDS) (https://www.seiscomp3.org/doc/applications/slarchive/SDS.html). Program *rt2ms* runs with the —F option which expects a text file containing the list of the RefTek input data to be processed. Finally, for every Julian day, the Unix command *cat* concatenates the reformatted mini-SEED hourly files into single-component daily files.

**Fig. 4:**
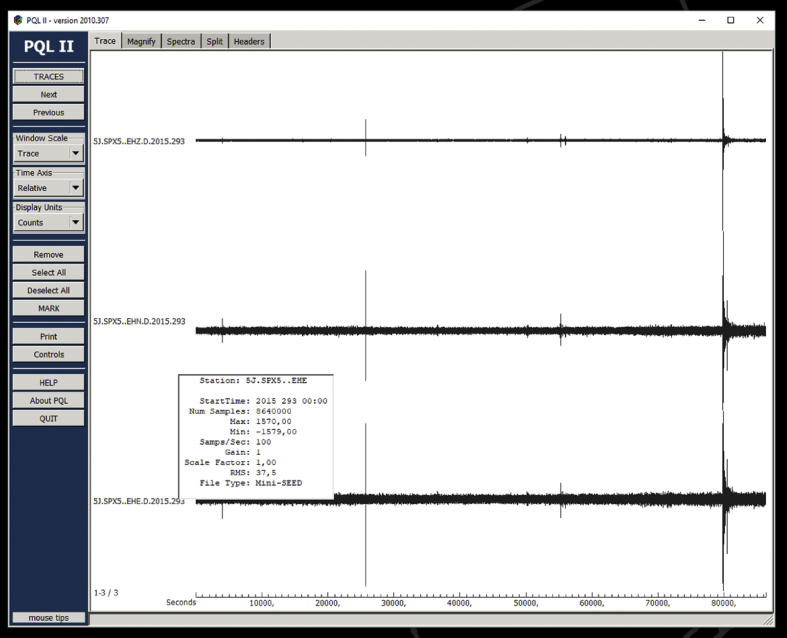
Screenshot of PQL program showing the SPX5 three-component recording for the day October 20, 2015 (Julian day 293). In the insert box the basic data that specify the single-component mini-SEED waveform file.

**Fig. 5:**
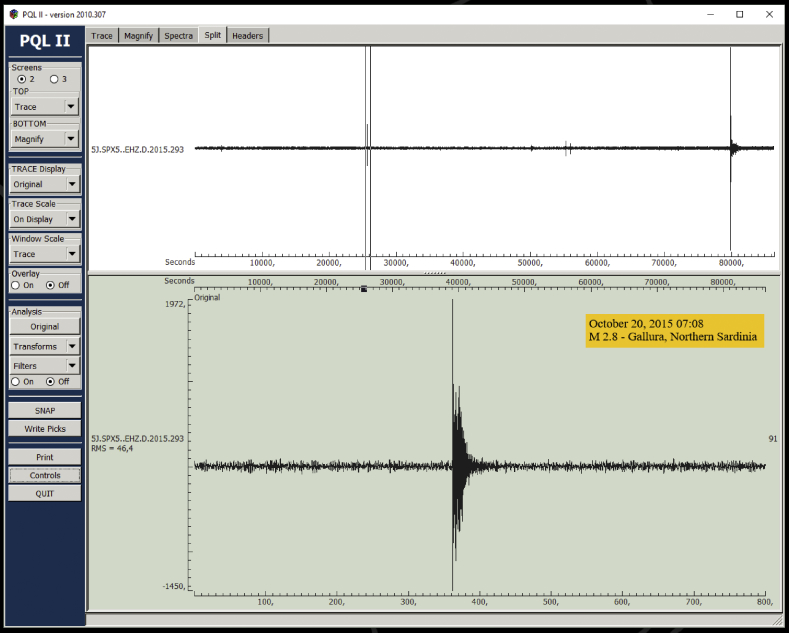
PQL screenshot showing the upper trace of Fig. 4 (vertical component) and, in the magnified portion, the recording of a local earthquake, magnitude M = 2.8, occurred North-West station SPX5 at about 30 km distance [1].

**Fig. 6:**
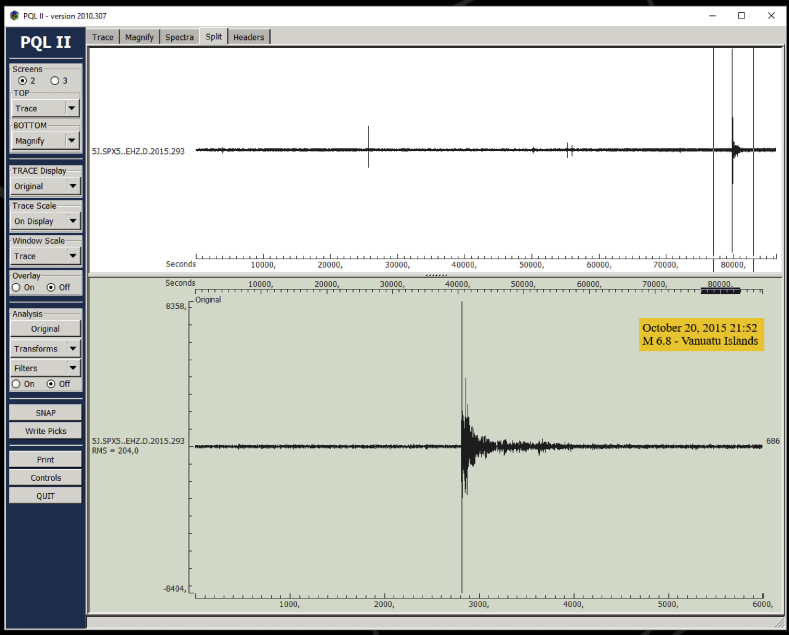
PQL screenshot showing the upper trace of Fig. 4 (vertical component) and, in the magnified portion, the recording of a distant, intermediate-depth earthquake (135 km depth), magnitude M = 6.8, occurred in the Vanuatu Islands region (https://earthquake.usgs.gov/earthquakes/eventpage).

Fig. 4 is a visualization of the mini-SEED files assembled for a day of raw data acquired by station SPX5. The screenshot captures the main display layout of the Passcal Quick Look version II (PQL II) program also available through the IRIS/PASSCAL download page. PQL is a program for viewing time-series data in different formats (MSEED, SEGY, SAC), zooming, filtering, computing fast Fourier spectra of seismic signals. It also allows arrival-time measurements (phase picking) and printing of results to a user-defined file. The data sample in Fig. 4 highlights the ambient noise level for the three ground motion components, data useful for estimating the seismic site response characteristics [11], and, more importantly, the presence of high-amplitude wave-trains likely related to earthquakes recording. The display layouts in Fig. 5, Fig. 6 (*Split* screens) illustrate the PQL interactive preview of such seismograms, which in the specific case were associated to a Sardinia local event and to a strong teleseism incoming from the Vanuatu Islands, respectively. These examples outline the pre-processing analysis that can be performed on the waveform dataset to check its quality and content. The latter can be explored in depth by using automatic earthquake detection and phase picking algorithms today increasingly sophisticated [12]. Some basic results from simple visual inspection of the SPAE record for the two events just above exemplified are reported in Table 4.

**Table 4:** Data from seismogram interpretation of the SPAE record collected on October 20, 2015. Error values indicate the estimated range of uncertainty measured by picking the earliest and the latest possible onset time for each reported phase [15]. First-motion polarities (C = compression, D = dilatation) are used in determining the focal mechanisms of earthquakes.

Station	MiniSEED file	Local earthquake				Teleseismic Event		
		Seismic phase	Arrival time	Error (s)	First-motion polarity	Seismic phase	Arrival time	Error (s)
SPX2	5J.SPX2..EHZ.D.2015.293	*Pg*	07:08:27.20	0.04	C	*PKPdf*	22:11:29.1	0.5
	5J.SPX2..EHN.D.2015.293	*Sg*	07:08:37.56	0.08				
	5J.SPX2..EHE.D.2015.293							
SPX5	5J.SPX5..EHZ.D.2015.293	*Pg*	07:08:17.58	0.01	D	*PKPdf*	22:11:28.1	0.3
	5J.SPX5..EHN.D.2015.293							
	5J.SPX5..EHE.D.2015.293	*Sg*	07:08:21.22	0.02				
SPX6	5J.SPX6..EHZ.D.2015.293	*Pg*	07:08:23.59	0.03	C	*PKPdf*	22:11:28.7	0.8
	5J.SPX6..EHN.D.2015.293	*Sg*	07:08:31.37	0.05				
	5J.SPX6..EHE.D.2015.293							

Additional *P*— and *S*-wave arrival times from local/regional sources are particularly important to obtain more precise hypocenter determination of the seismic activity affecting the Sardinia-Corsica block. Indeed, the seismicity of the region is often characterized by poorly constrained solutions (e.g. large *rms* misfits and azimuthal gaps, focal depths uncontrolled), or only partially detected by the existing networks given their current coverage. Accurate phase arrivals can also be used to determine reliable 1D crustal seismic velocity models. Information on this topic are limited basically to the continental margins of the microplate, and comes from the several reflection/refraction data collected during the last decades [13]. Finally, it is worth mentioning that in Sardinia many quarries and mines are still active, the former especially in the northern (Gallura granite district) and central-eastern (Orosei marble district) parts of the island where the temporary network concentrated (Fig. 1). The SPAE waveform dataset can therefore also be scanned for blasts record, and methods comparing *S*— to *P*-wave amplitude ratios, *P*-wave spectra, and time-frequency analysis [14] can be implemented to distinguish them from natural seismicity.

The whole SPAE seismic dataset is allocated on the EIDA platform under the temporary network deployment 5J_2014. 5J is the network code given by FDSN to the passive experiment (http://www.fdsn.org/networks/detail/5J_2014/). Data are open access and can be downloaded using the ORFEUS Data Center WebDC3 Web Interface (orfeus-ue.org/webdc3/).
