# Supplementary material for: The seismic waveform dataset of the Sardinia Passive Array Experiment (SPAE)
Source: Data Brief. 2019 Apr 17;24:103927. doi: 10.1016/j.dib.2019.103927 (PMC6514363; doi:10.1016/j.dib.2019.103927)
Supplement: Multimedia component 1 [file mmc1.docx]

**Appendix A. Supplementary material**

PDF Analysis of background seismic noise at SPAE stations


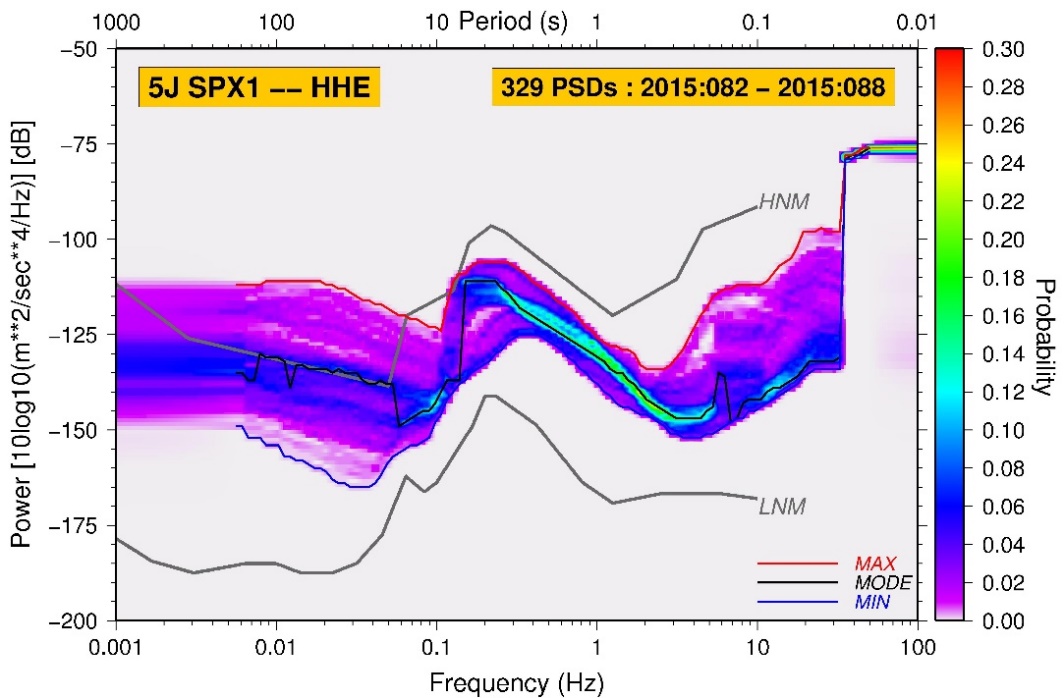


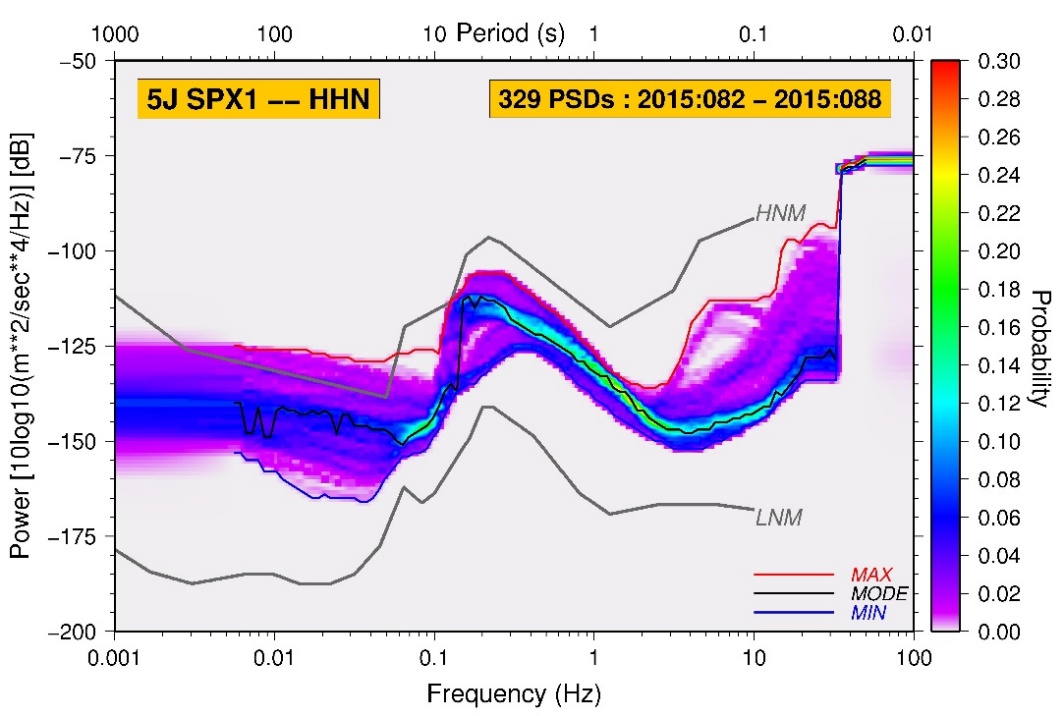


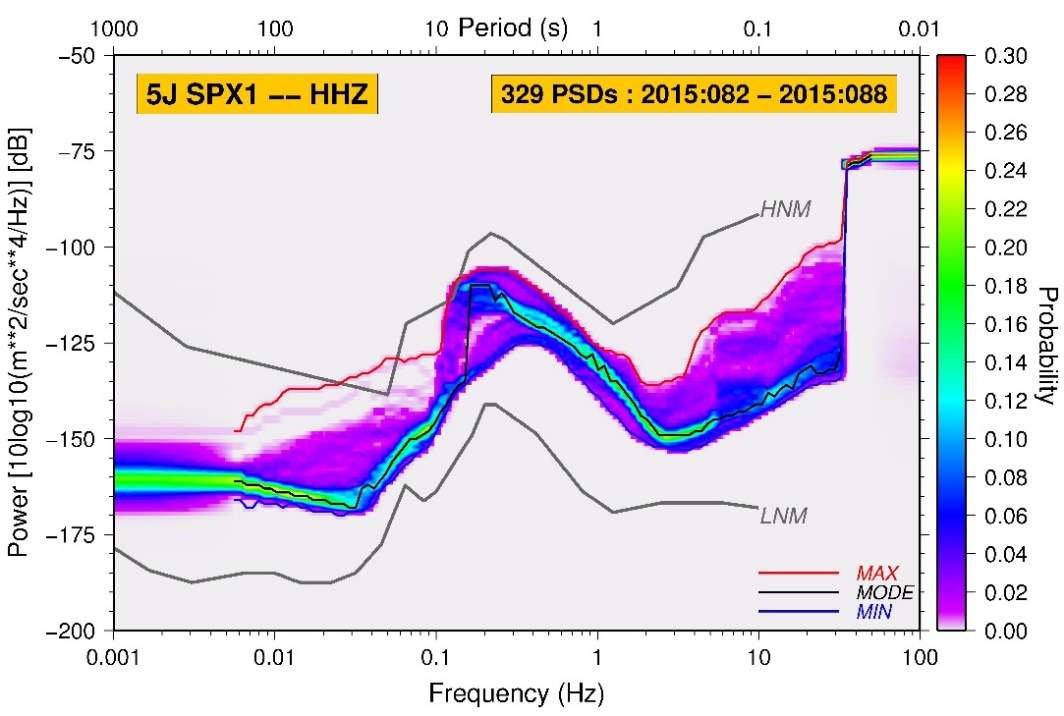


**Figure 1** PDF seismic noise analysis for station SPX1 - March 23-29, 2015.


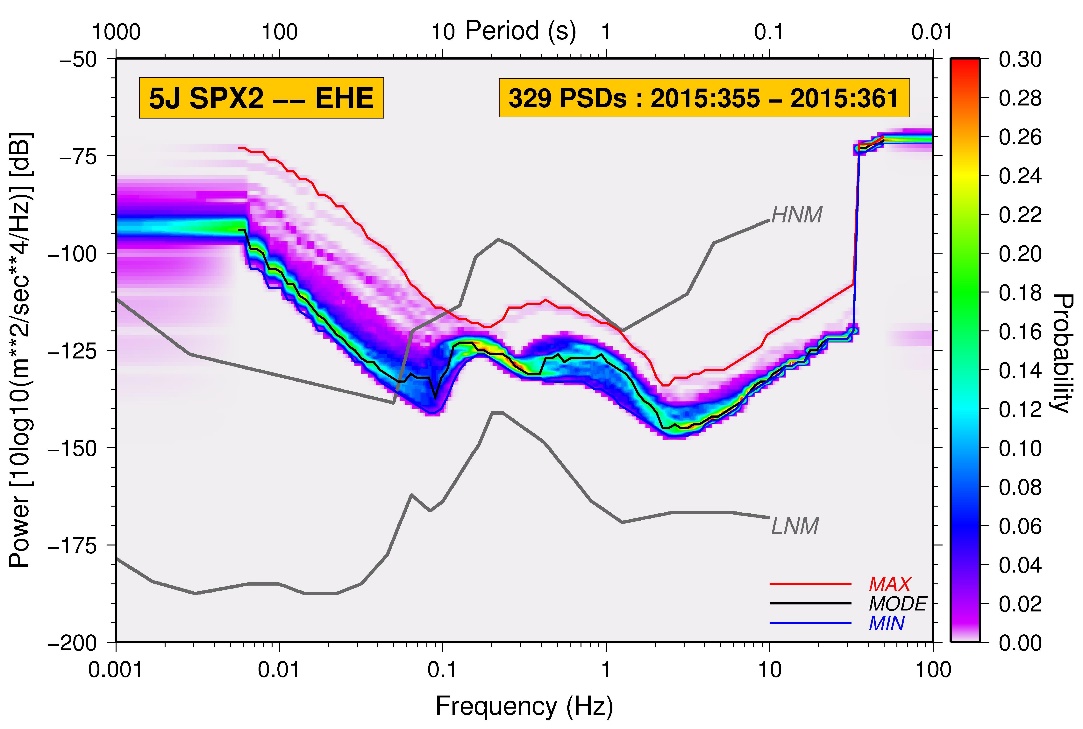


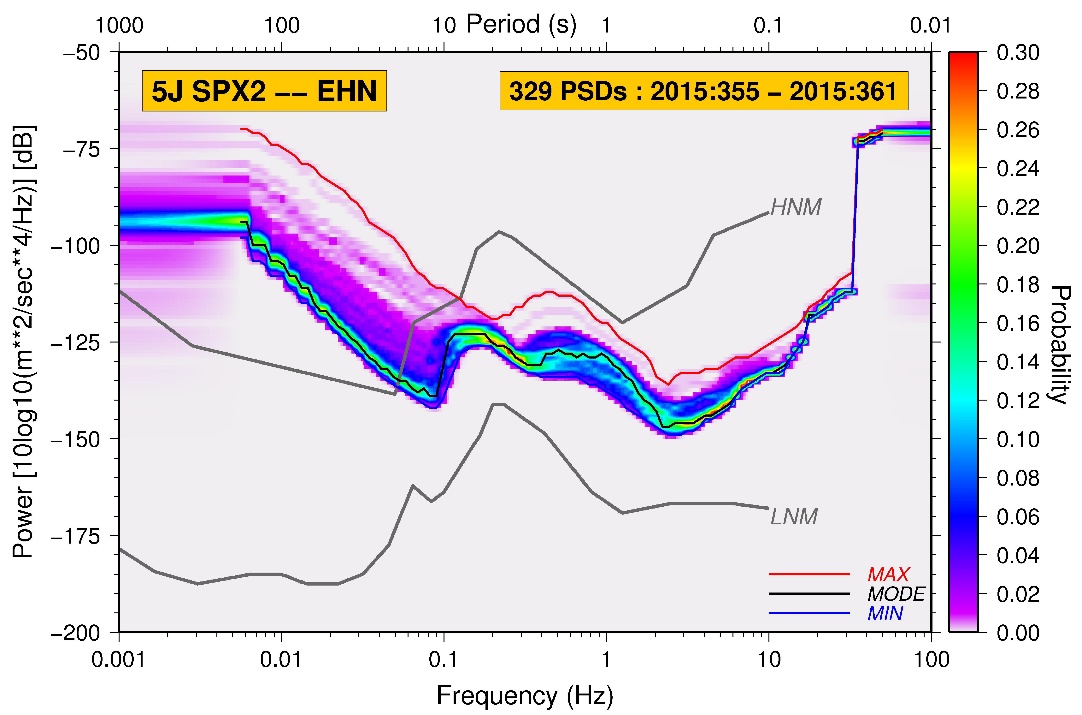


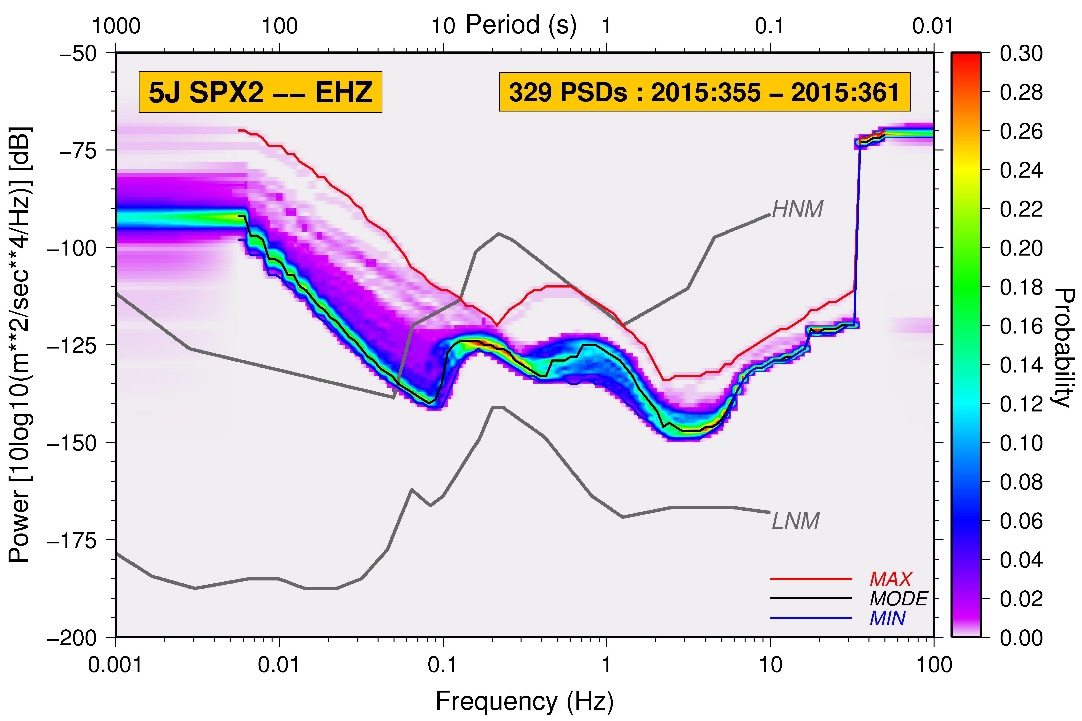


**Figure 2** PDF seismic noise analysis for station SPX2 - December 21-27, 2015.


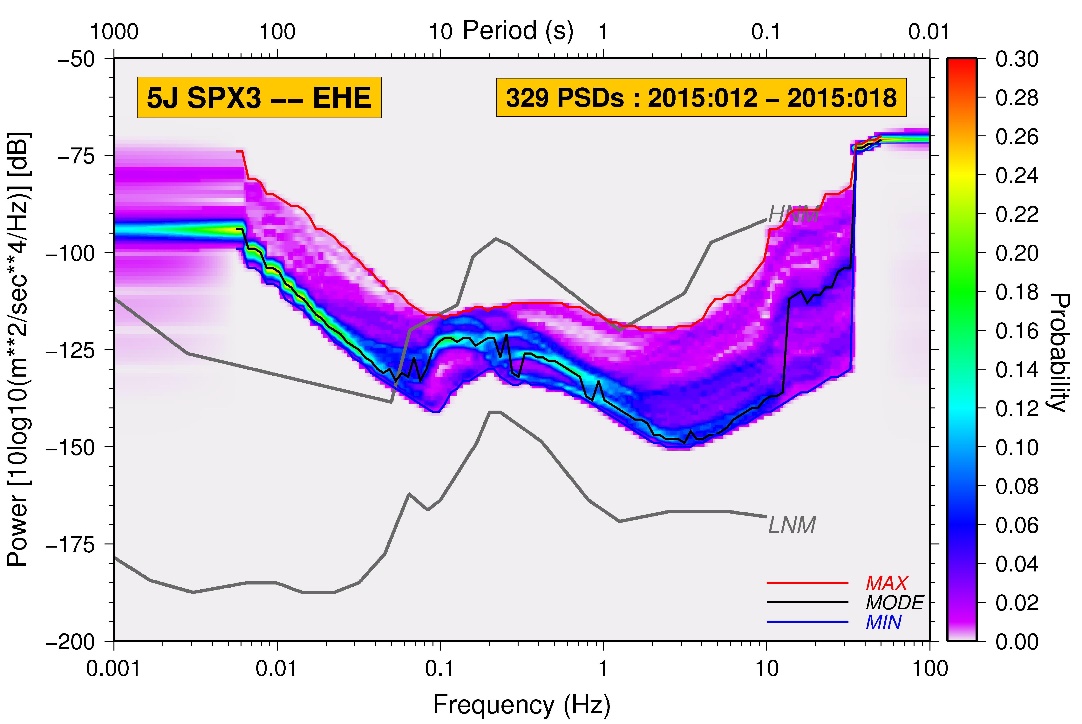


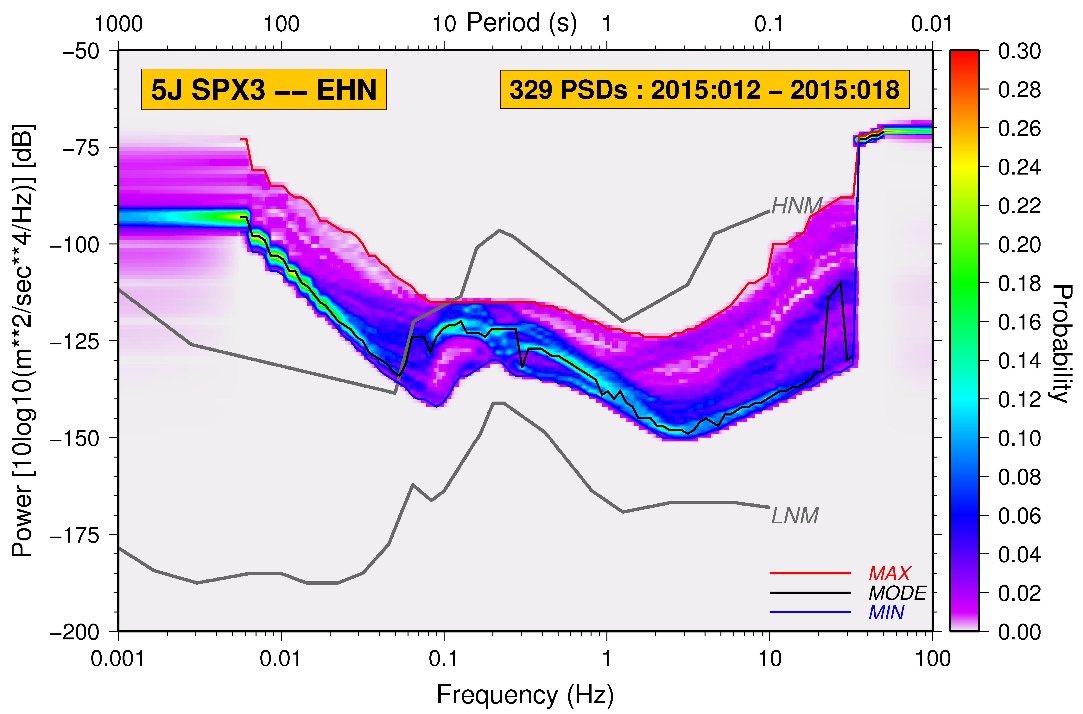


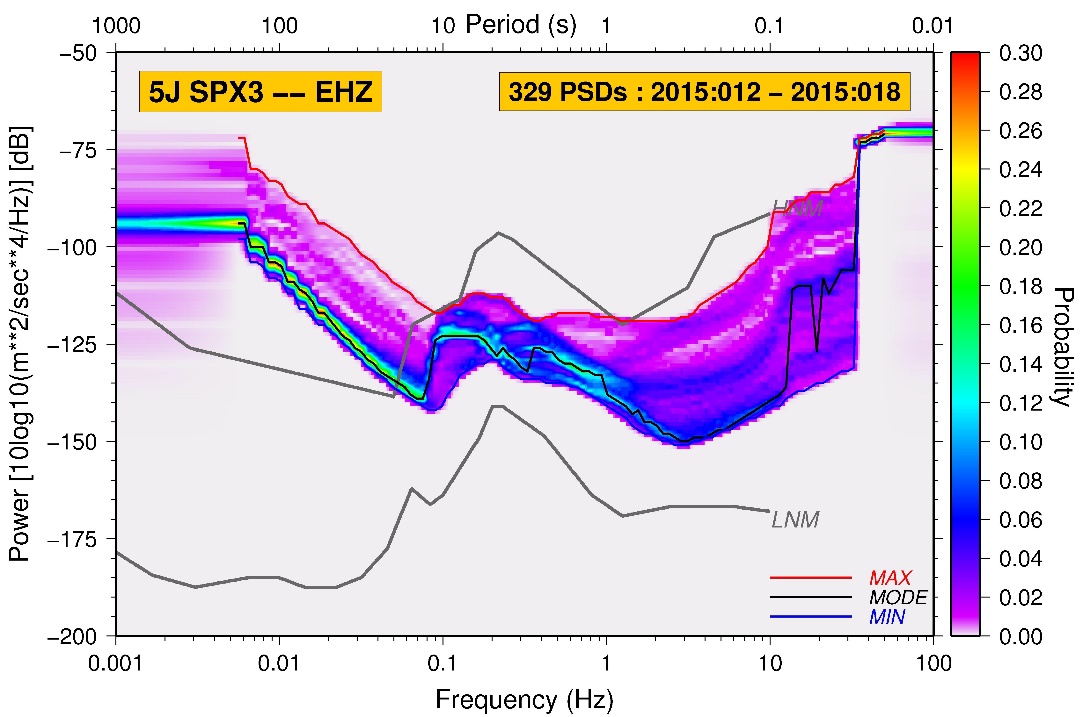


**Figure 3** PDF seismic noise analysis for station SPX3 - January 12-18, 2015.


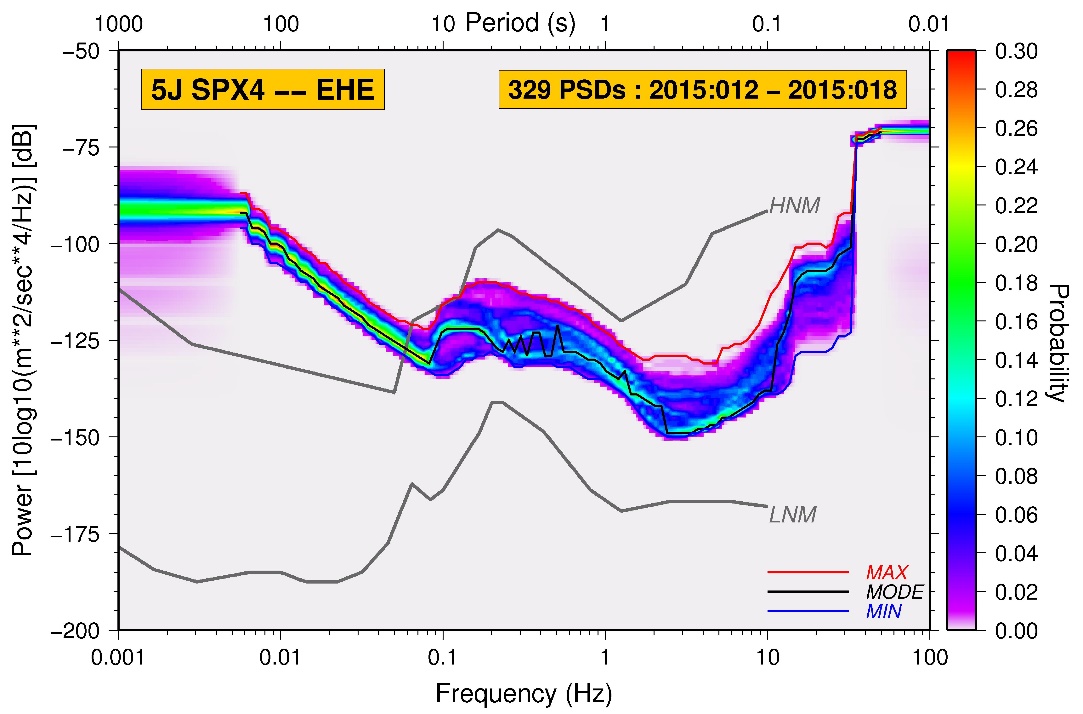


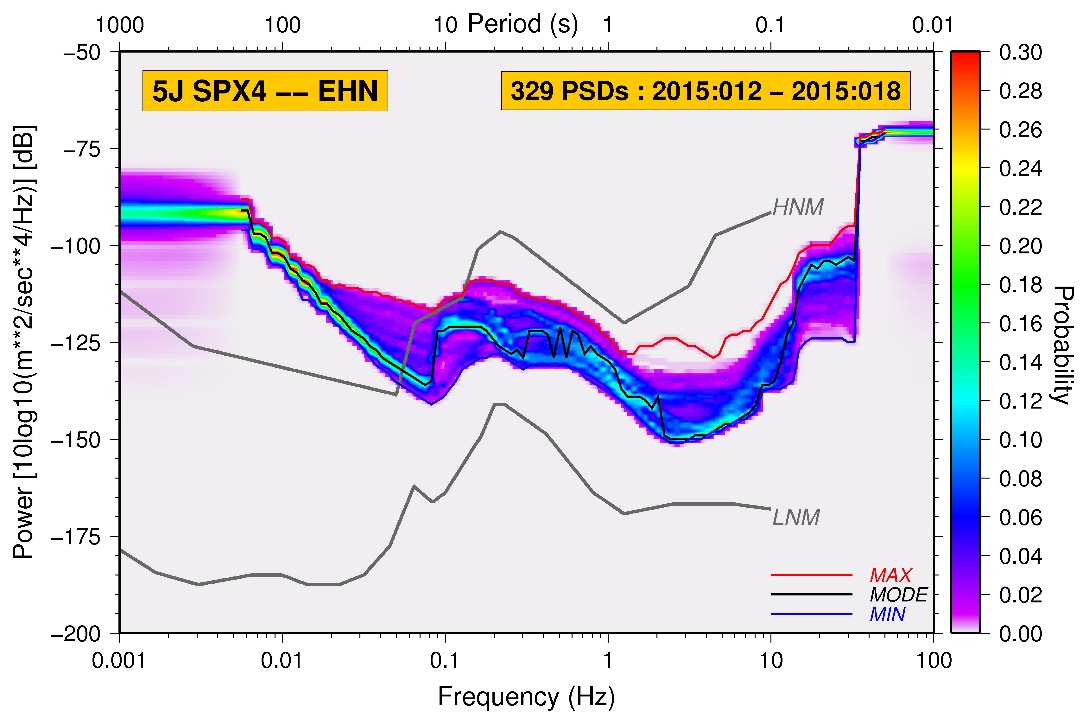


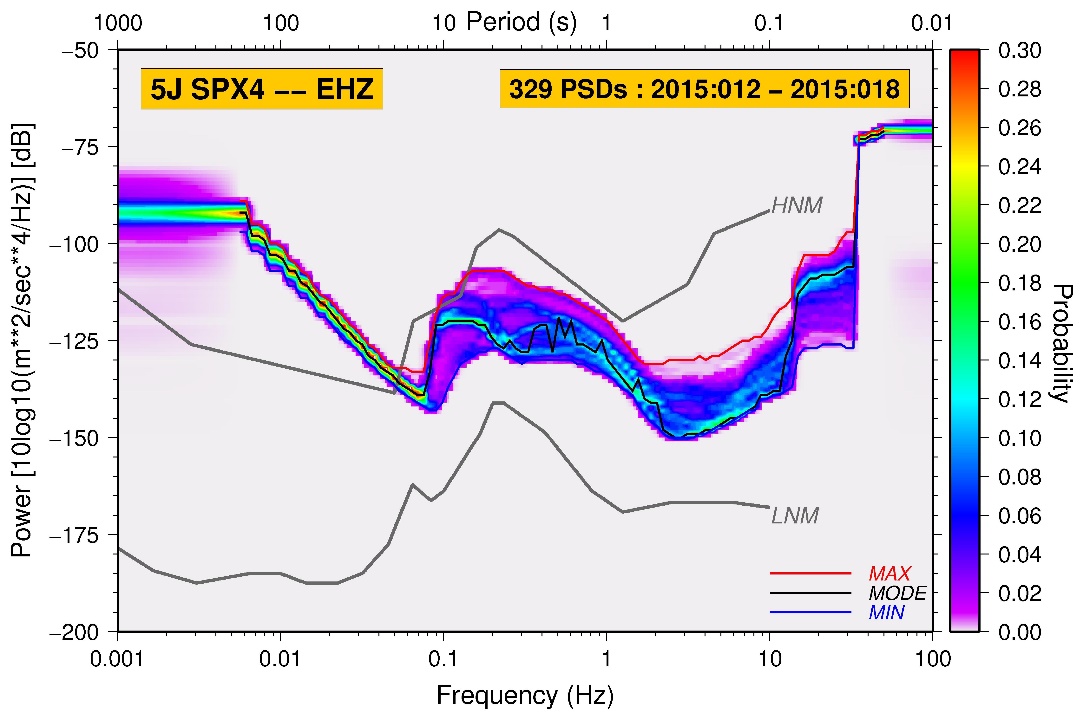


**Figure 4** PDF seismic noise analysis for station SPX4 - January 12-18, 2015.


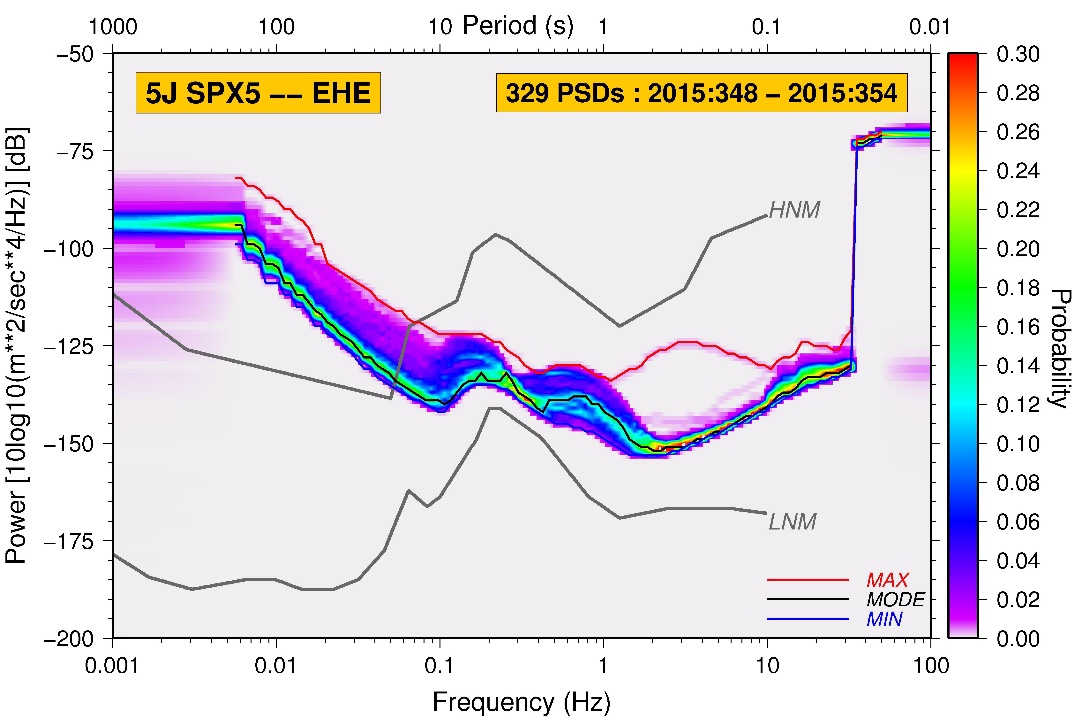


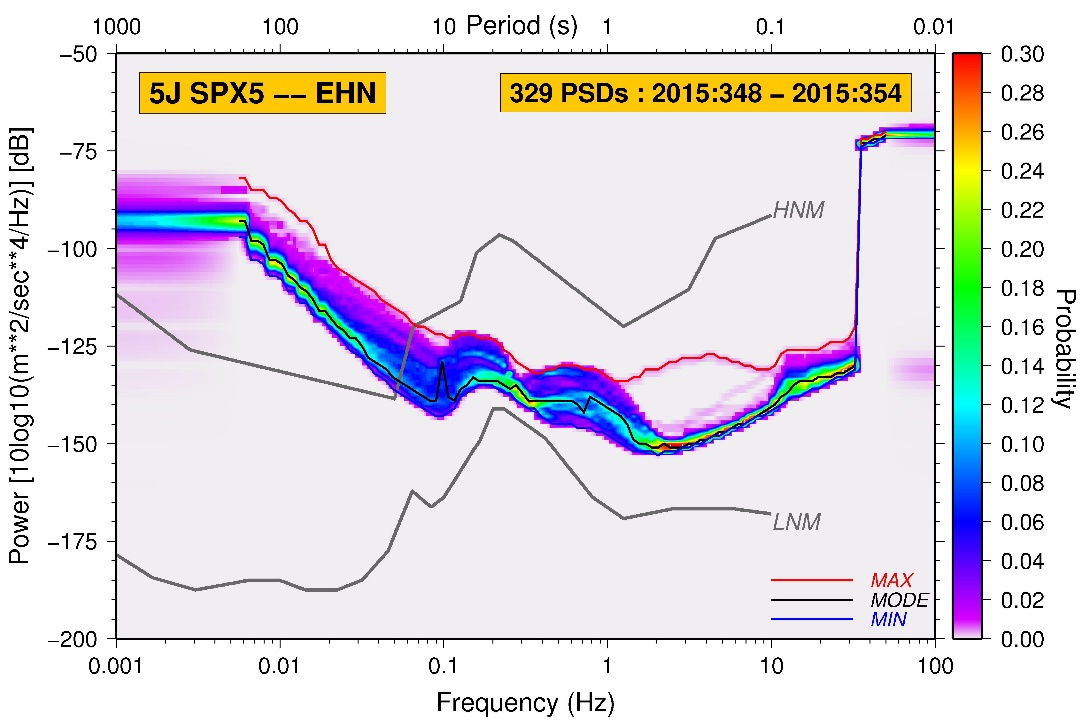


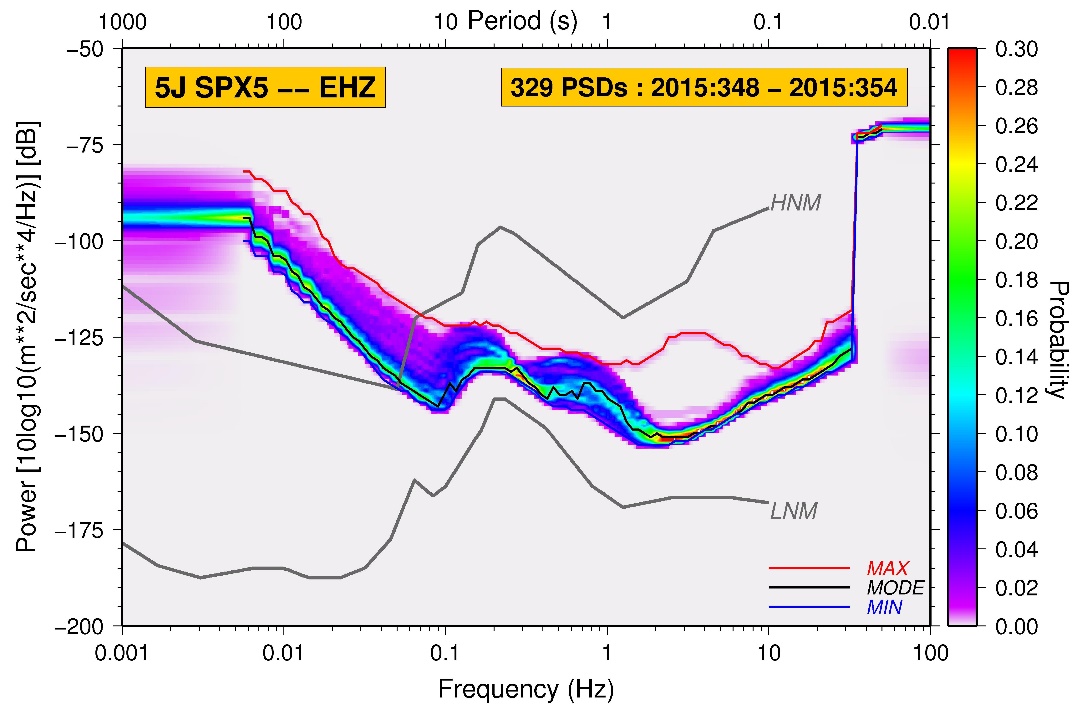


**Figure 5** PDF seismic noise analysis for station SPX5 - December 14-20, 2015.


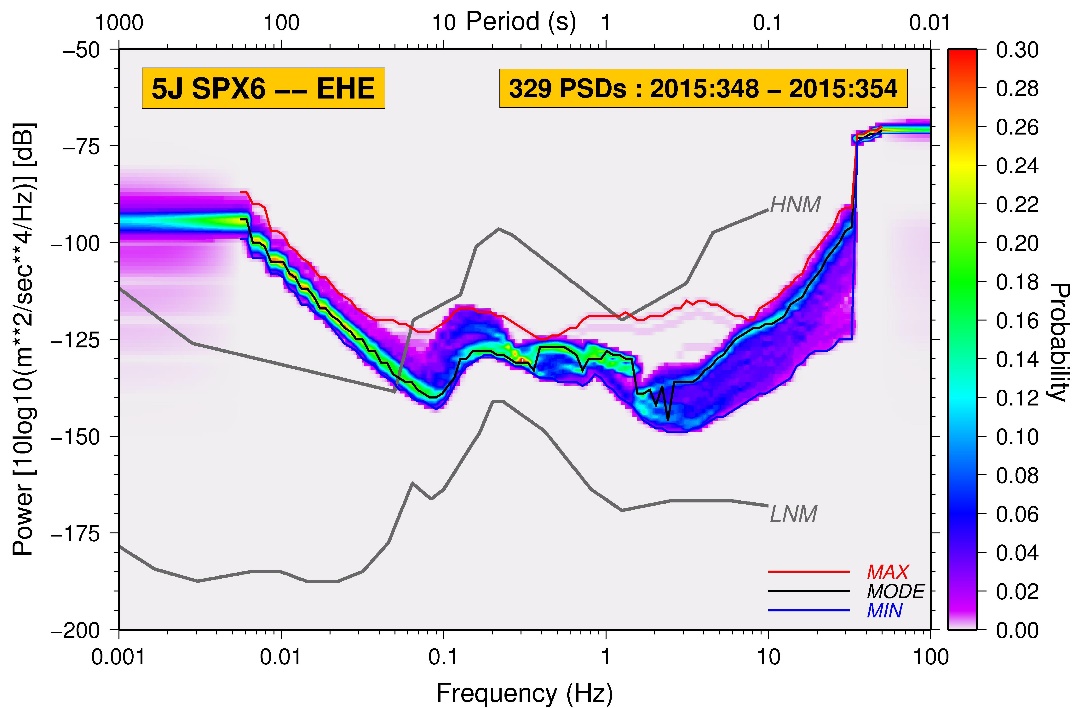


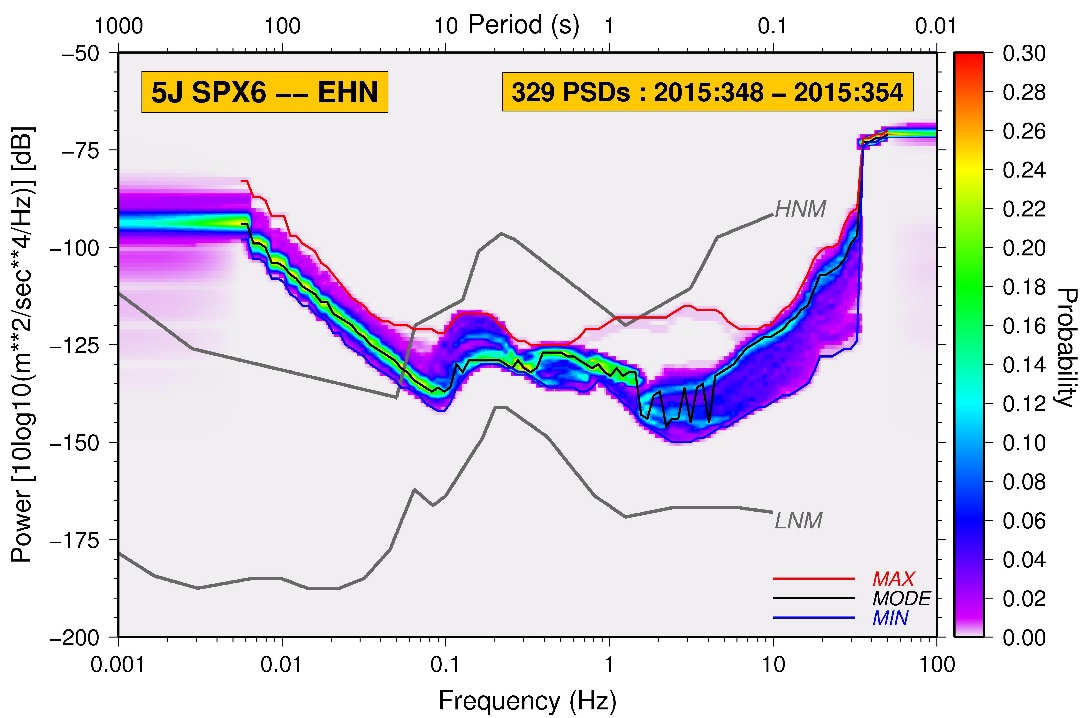


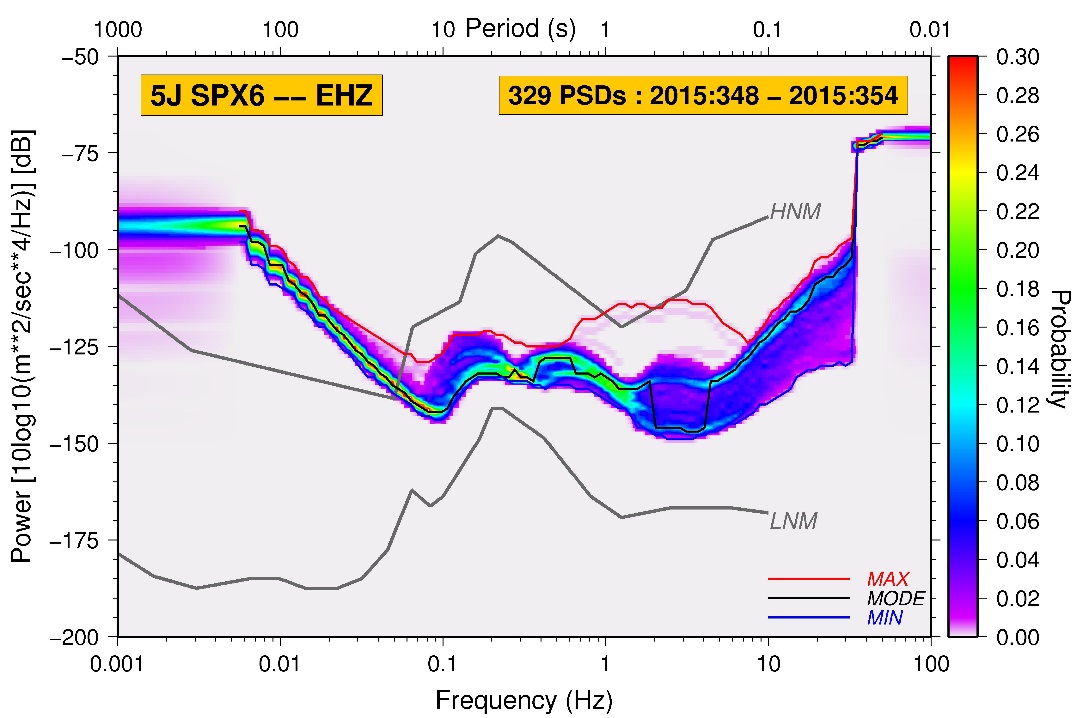


**Figure 6** PDF seismic noise analysis for station SPX6 - December 14-20, 2015.


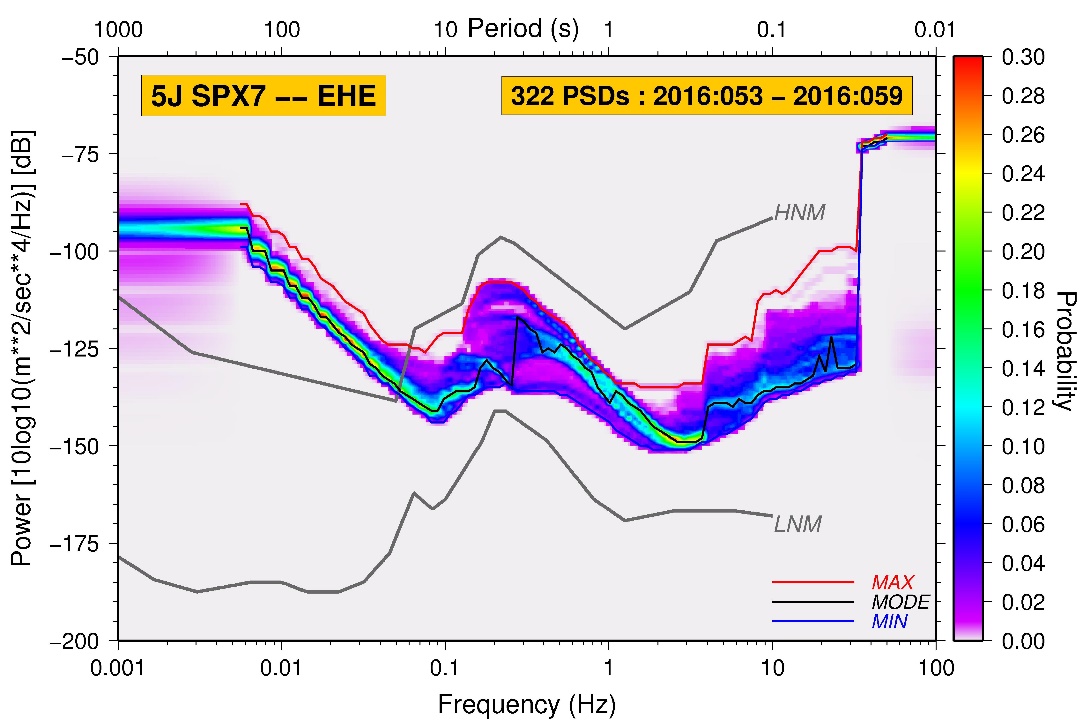


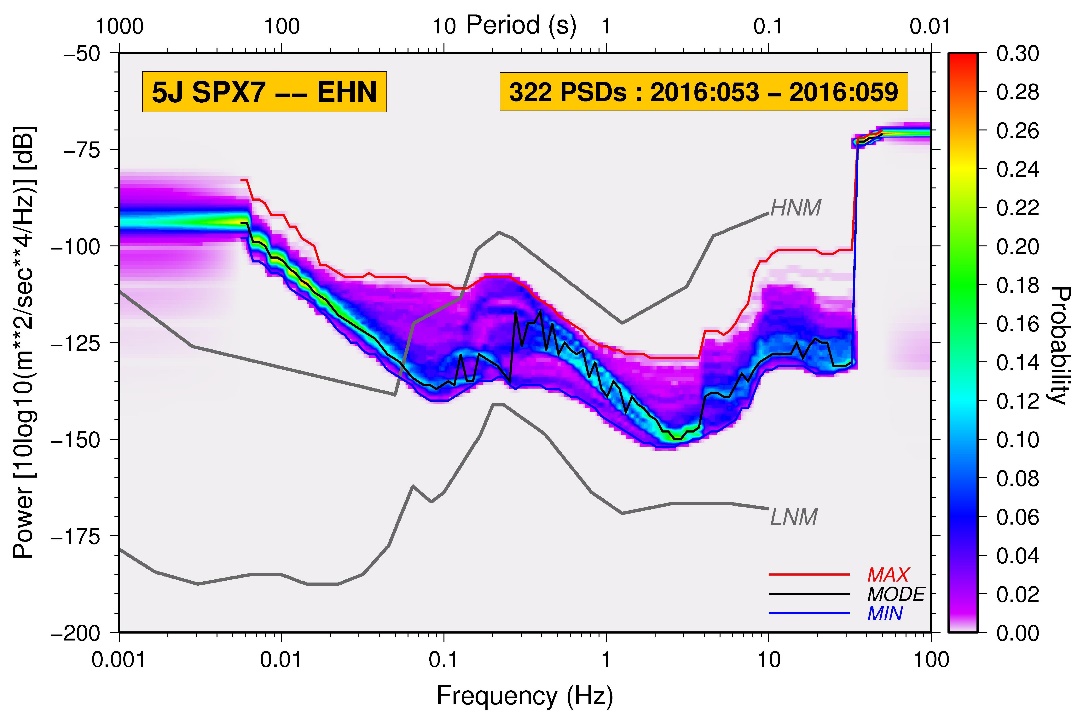


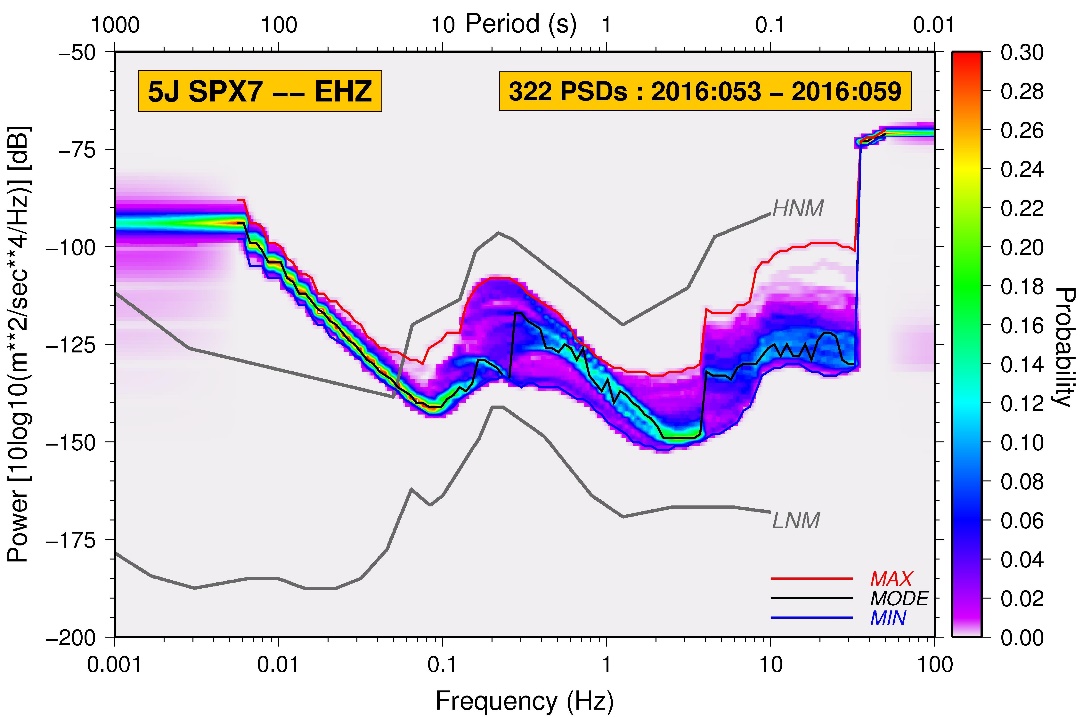


**Figure 7** PDF seismic noise analysis for station SPX7 - March 22-28, 2016.


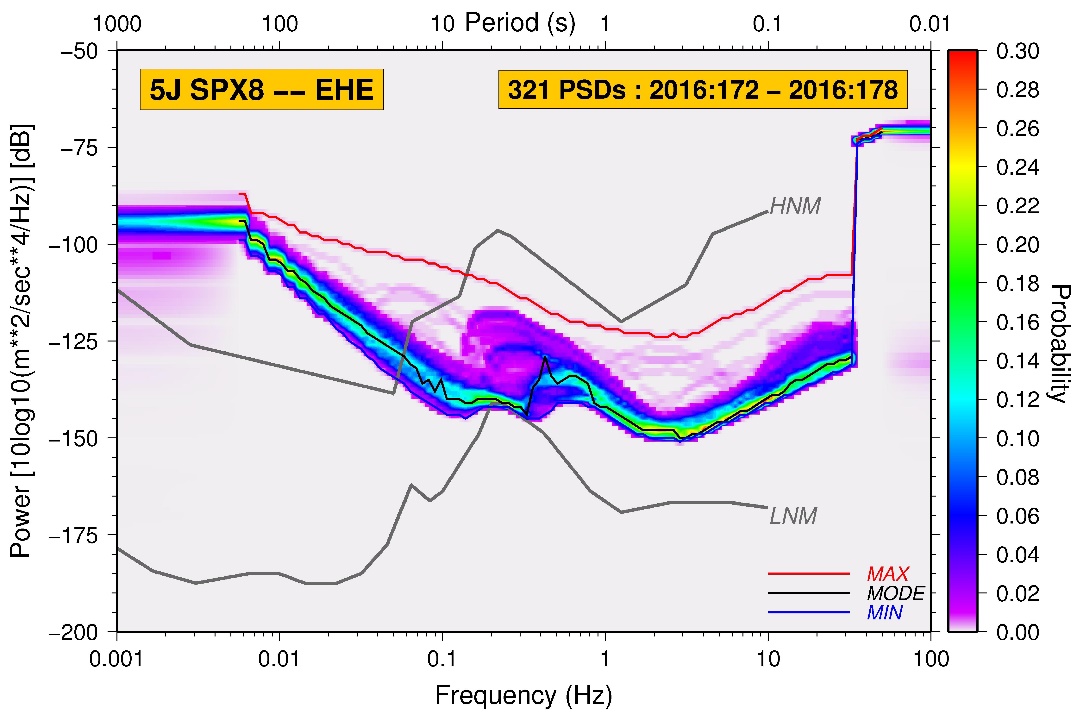


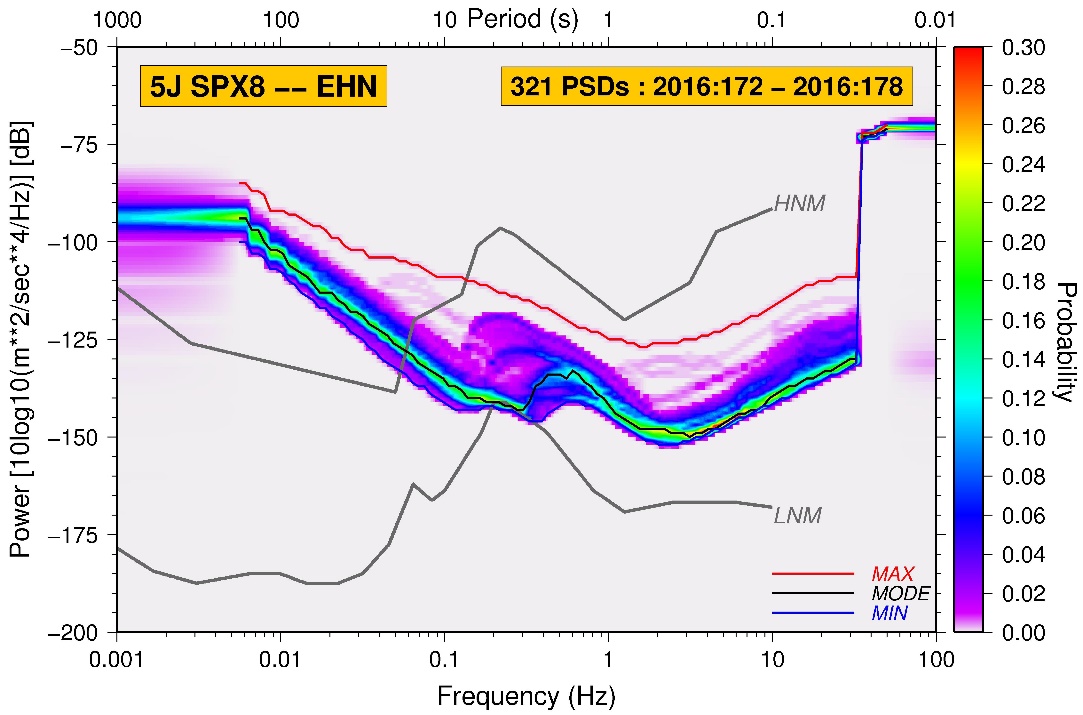


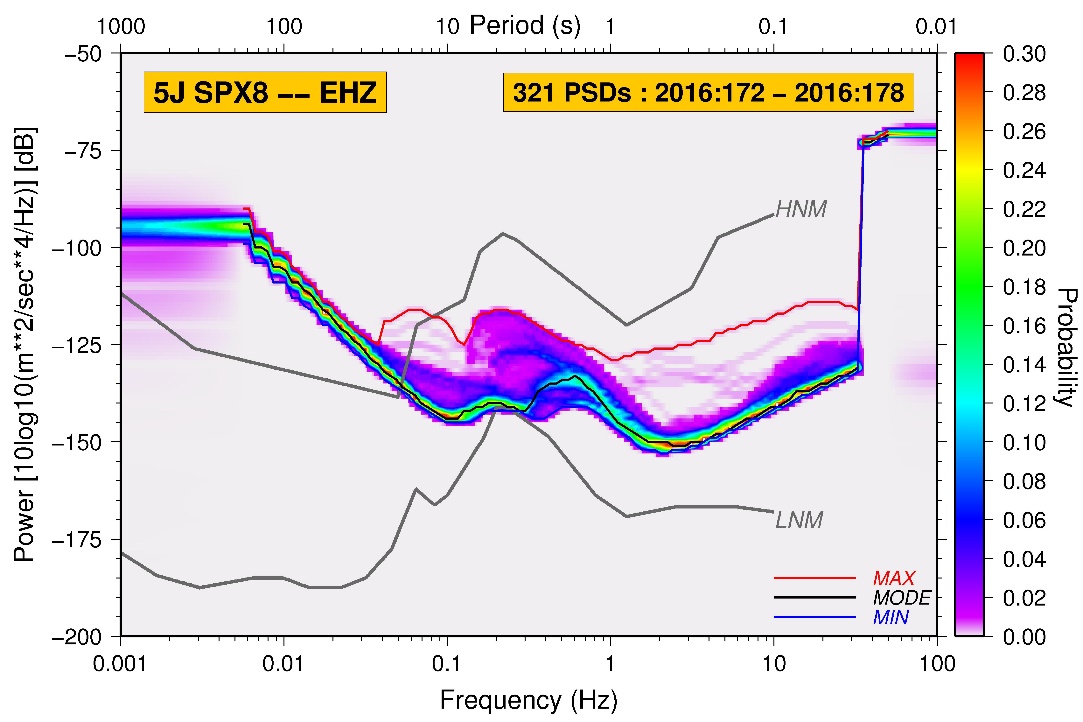


**Figure 8** PDF seismic noise analysis for station SPX8 – June 20-26, 2016.
