# Supplementary material for: The seismic waveform dataset of the Sardinia Passive Array Experiment (SPAE)
Source: Data Brief. 2019 Apr 17;24:103927. doi: 10.1016/j.dib.2019.103927 (PMC6514363; doi:10.1016/j.dib.2019.103927)
Supplement: Multimedia component 2 [file mmc2.docx]

**Appendix B. Supplementary material**

Unix script used to generate mini-SEED daily files from RefTek RT130 raw data.

#!/bin/bash

#

# Script: MS_SPAE

#

# Station: SPX1

# Year: 2014

# Days: 198 - 365

# Channels: EHZ, EHN, EHE

#

MSDIRZ=/home/SPAE/2014/5J/SPX1/EHZ.D

MSDIRN=/home/SPAE/2014/5J/SPX1/EHN.D

MSDIRE=/home/SPAE/2014/5J/SPX1/EHE.D

#

MSZ=5J.SPX1..EHZ.D.2014.

MSN=5J.SPX1..EHN.D.2014.

MSE=5J.SPX1..EHE.D.2014.

#

rt2ms -F RTDATA_SPX1_2014 -p SPX1_rt2ms.par -R

#

ls -d R*.01 > Rdays.list

#

while read RDIR

do

JDAY=${RDIR:1:3}

MSFZ=$MSZ$JDAY

MSFN=$MSN$JDAY

MSFE=$MSE$JDAY

#

cat $RDIR/*1.1.m > $MSFZ

cat $RDIR/*1.2.m > $MSFN

cat $RDIR/*1.3.m > $MSFE

#

mv $MSFZ $MSDIRZ/.

mv $MSFN $MSDIRN/.

mv $MSFE $MSDIRE/.

#

rm -fR $RDIR

#

done < Rdays.list

#
