# Supplementary material for: The seismic waveform dataset of the Sardinia Passive Array Experiment (SPAE)
Source: Data Brief. 2019 Apr 17;24:103927. doi: 10.1016/j.dib.2019.103927 (PMC6514363; doi:10.1016/j.dib.2019.103927)
Supplement: Multimedia component 3 [file mmc3.docx]

The author declare no conflict of interest in this article.

'Conflicts of interest: none'.
